# Case Report: Perioperative Management of a Patient with Glycogen Storage Disease Type IXd

**DOI:** 10.70352/scrj.cr.25-0239

**Published:** 2025-09-04

**Authors:** Koto Kawata, Hajime Otsu, Qingjiang Hu, Yasuo Tsuda, Yoshihiro Nagao, Yusuke Yonemura, Takaaki Masuda, Tomoharu Yoshizumi, Koshi Mimori

**Affiliations:** 1 Department of Surgery, Kyushu University Beppu Hospital, Beppu, Oita, Japan; 2 Department of Surgery and Science, Graduate School of Medical Sciences, Kyushu University, Fukuoka, Fukuoka, Japan; 3 Department of Surgery, Breast Surgical Oncology, Kochi Medical School, Kochi University, Nankoku, Kochi, Japan

**Keywords:** glycogen storage disease type IXd, postoperative management, rhabdomyolysis

## Abstract

**INTRODUCTION:**

Glycogen storage disease type IX (GSD type IX) is caused by a deficiency in phosphorylase b kinase (PHK) and is classified into hepatic (IXa–c) and muscular (IXd) subtypes. GSD type IXd leads to exercise intolerance, rhabdomyolysis, and myoglobinuria owing to impaired glycogen breakdown. It is a rare and mild metabolic disorder, with only 19 reported cases of *PHKA1* mutations. To the best of our knowledge, this is the 1st report on the perioperative management of a patient with GSD type IXd.

**CASE PRESENTATION:**

A 61-year-old male presented with a right inguinal hernia requiring surgical repair. He had experienced muscle weakness since the age of 53, which progressed to severe neck muscle atrophy by the age of 58. Genetic testing confirmed a *PHKA1* mutation, leading to the diagnosis of GSD type IXd. He had previously undergone multiple surgeries without any complications. Given his underlying muscle weakness, totally extraperitoneal (TEP) inguinal hernia repair was performed to minimize postoperative pain and muscle damage. Postoperative monitoring revealed no rhabdomyolysis or myoglobinuria, and the patient was discharged without complications on POD 7.

**CONCLUSIONS:**

We successfully managed a patient with GSD type IXd perioperatively, without complications. Although this disease can cause rhabdomyolysis, the symptoms are often mild and may remain undiagnosed. Therefore, in patients with muscle weakness or elevated creatine kinase levels, careful surgical planning and perioperative monitoring are essential.

## Abbreviations

AST
: aspartate aminotransferase
BMI
: body mass index
CK
: creatinine kinase
CRP
: C-reactive protein
GSD
: glycogen storage disease
LDH
: lactate dehydrogenase
PHK
: phosphorylase b kinase
TEP
: totally extraperitoneal
US
: ultrasonography

## INTRODUCTION

GSD type IX is caused by a deficiency in PHK and comprises subtypes IXa, IXb, IXc, and IXd. PHK, a key enzyme involved in glycogen breakdown, phosphorylates glycogen phosphorylase, thereby inducing a change from its inactive to the active form. PHK comprises 4 subunits with a stoichiometry of α4β4γ4δ4. The γ subunit is catalytic, the α and β subunits are regulatory, and the δ subunit is identical to calmodulin. The α subunit has 2 different isoforms that show tissue-specific expression in the muscle (αM) and liver (αL).^1)^

Subtypes IXa–c are hepatic and are associated with symptoms such as hypoglycemia, whereas subtype IXd is muscular and is associated with exercise intolerance, rhabdomyolysis, and myoglobinuria.^2)^ The IXd subtype can be caused by mutations in *PHKA1* (encoding the αM subunit of PHK) on the X chromosome, as well as mutations in *PHKG1* (encoding the γ subunit). Both conditions are rare and result in mild metabolic disorders.^3)^ To date, only 19 cases of GSD type IXd with *PHKA1* mutations have been reported, and these reports have focused on *PHKA* mutations and clinical findings.^1–15)^

To the best of our knowledge, this is the 1st report on the perioperative management of a patient with GSD type IXd.

## CASE PRESENTATION

A 61-year-old male was referred to our hospital for surgery for a right inguinal hernia with pain and right inguinal bulging.

He had experienced post-exercise muscle weakness since approximately the age of 53 years. By the age of 58 years, his neck muscles had become so weak that he could not support his head without a cervical collar. He had no limitation of movement but showed significant muscle weakness and muscle atrophy in the neck, muscle weakness and muscle atrophy in the proximal muscles of the extremities, and muscle atrophy in the trunk muscles, including the paraspinal muscles. Blood tests at the referral hospital revealed elevated CK levels, and myositis was suspected. Despite treatment with steroids and immunosuppressants, no improvement was observed. All tests for myositis-specific and immune-related antibodies yielded negative results. Muscle biopsy revealed glycogen accumulation under the muscle fibers. Subsequent genetic testing revealed a deletion of 17 bases in the exon region of *PHKA1*, and GSD type IXd was diagnosed. Thereafter, blood tests were performed regularly on an outpatient basis. When CK elevation was detected, the patient was admitted to the hospital for bed rest. At the age of 60 years, he was hospitalized once for a CK elevation of 1330 U/L but was discharged after he recovered on bed rest.

His height was 163.5 cm, body weight was 60.2 kg, and BMI was 22.5 kg/m^2^. He had undergone surgery for stomach cancer at the age of 35 years, bowel obstruction at the age of 50 years, and left inguinal hernia at the age of 57 years, without any problems. There was no family history of muscular diseases, including GSD. Additionally, he reported no history of alcohol consumption or smoking and was not taking any statins.

**Fig. 1:**
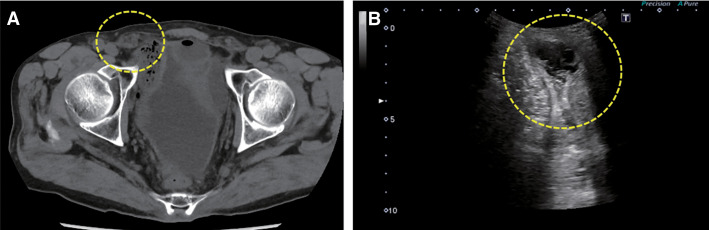
CT shows no hernia (**A**), but US shows an external inguinal hernia with small bowel prolapse in the right inguinal region (**B**). US, ultrasonography

The AST and LDH levels were 33 U/L (normal range: 13–30 U/L) and 376 U/L (normal range: 124–222 U/L), respectively. The levels of serum CK and CRP were 598 U/L (normal range: 59–248 U/L) and 0.61 mg/dL (normal range: ≤0.14 mg/dL), respectively. We observed about a 30 mm swelling in the right inguinal region; however, manual reduction was possible. CT revealed no hernia (**Fig. 1A**); however, US revealed an external inguinal hernia with small bowel prolapse in the right inguinal region (**Fig. 1B**). On preoperative examination, no abnormalities were observed in the cardiac or respiratory function.

Inguinal hernia repair was performed at the patient’s request using the TEP approach, due to the symptomatic hernia. The prosthetic mesh was fixed in the usual manner using tacks. To prevent postoperative neuralgia, the mesh was secured at safe anatomical landmarks—including the pubis, Cooper’s ligament, rectus abdominis, and transversus abdominis—using the minimum number of tacks necessary.

The operation lasted 45 min with minimal blood loss and was completed without any complications. The usual surgical devices were used; however, anapeine was applied to the preperitoneal space as a local anesthetic to reduce postoperative pain. On POD 1, blood tests showed AST and LDH levels of 30 and 318 U/L, respectively. The CK and CRP levels were 425 U/L and 0.14 mg/dL, respectively, with no worsening compared with the preoperative levels. On POD 4 the levels of AST and LDH were 21 and 276 U/L, respectively. The CK level further improved to 291 U/L. A urinalysis performed on the same day as the blood test showed no myoglobinuria (**Table 1**). The patient was discharged without complications on POD 7.

**Table 1:** Blood and urine test results

Day	AST (U/L)	LDH (U/L)	CK (U/L)	CRP (mg/dL)	Occult hematuria	Red blood cells in urine
Preoperative	33	376	598	0.61	+/–	–
POD 1	30	318	425	0.14	–	–
POD 4	21	276	291	4.57 (cystitis)	–	–

AST, aspartate aminotransferase; CK, creatinine kinase; CRP, C-reactive protein; LDH, lactate dehydrogenase

## DISCUSSION

Patients with GSD type IXd may experience painful muscle spasms during exercise, exercise intolerance, rhabdomyolysis, and myoglobinuria.^2)^ In this condition, the abnormal *PHK* causes reduced activity, which makes it difficult for the muscle to break down glycogen and produce ATP, thus preventing the muscle from producing energy when a heavy load is applied, leading to muscle damage. The Japanese Society for Inherited Metabolic Diseases and previous reviews^16)^ have identified no fundamental cure for this disease. Acute treatments include high-volume infusions, hyperkalemia management, urinary alkalinization therapy, and hemodialysis. Preventive measures include isometric exercise and avoidance of drugs such as succinylcholine and statins.

In the present case, preoperative muscle weakness was observed, which interfered with the daily activities. Therefore, minimizing further muscle damage was necessary. This patient also presented with a symptomatic (painful) inguinal hernia. If conservative observation had been chosen, there was a concern that pain-induced muscle tension could lead to muscle injury, potentially exacerbating the underlying GSD. In addition, there was a risk of incarceration, which could necessitate emergency surgery. Therefore, the benefits of elective surgical intervention were considered to outweigh the risks. After explaining the risks associated with the surgical procedure and anesthesia, the patient elected to proceed with surgical treatment. To that end, in consultation with specialists from other departments, we examined the surgical techniques and perioperative management strategies to reduce muscle damage caused by muscle contraction.

Currently, there are no reports on the use of general anesthesia for GSD type IXd. In hepatic-type GSD, preoperative and intraoperative intravenous glucose administration is sometimes required due to the risk of fasting-induced ketosis and hypoglycemia. However, this patient had the myopathic type of GSD, and there were no issues with blood glucose levels; therefore, glucose administration was not necessary. In myopathic GSD, impaired ATP production in muscles can lead to reduced metabolic capacity, potentially resulting in delayed breakdown, excretion, and reuptake of muscle relaxants, thereby prolonging their effects. Additionally, previous reviews^16)^ have reported the possibility of malignant hyperthermia and rhabdomyolysis in such patients. Therefore, the use of succinylcholine is avoided, and even non-depolarizing muscle relaxants should be used with caution. The patient had muscle weakness due to the GSD and cervical spondylotic myelopathy, which restricted neck extension. Therefore, intubation and extubation could be difficult under general anesthesia. Furthermore, as mentioned earlier, the risks of prolonged muscle relaxation and malignant hyperthermia owing to the use of muscle relaxants are high. Considering these risks, an anterior approach under local anesthesia was considered. However, severe postoperative pain can lead to further muscle damage. Laparoscopic surgery reportedly causes less chronic pain than the anterior approach.^17)^ Therefore, we decided to use the TEP method, which is routinely performed. As the surgeons were highly experienced in this technique, and a shorter operative time and relatively less postoperative pain were expected.

We recommend that each institution employ the surgical and anesthetic methods with which they are most familiar. This is because, when comparing the risk of prolonged general anesthesia potentially leading to difficulty with extubation versus the risk of postoperative pain, the former is considered to pose a greater threat to life. There are currently no reports specifically addressing perioperative management in patients with this disease, and the risks and benefits of general versus local anesthesia remain largely unknown.

Preoperatively, blood tests were conducted to confirm the patient’s control values. Additionally, information regarding the disease was shared with physicians from other departments and co-medical staff within the hospital. Postoperatively, we regularly assessed the degree of pain through interviews and monitored the CK levels via blood tests to check for rhabdomyolysis, as well as evaluated kidney damage by checking for myoglobinuria via urinalysis, which extended the hospital stay to 7 days, slightly longer than usual. If CK elevation or myoglobinuria was observed, we planned to implement rest and massive fluid infusion for washout in accordance with the guidelines of the Japanese Society for Inherited Metabolic Diseases. Fortunately, in this case, the postoperative CK elevation was within an acceptable range, and no rhabdomyolysis or myoglobinuria was observed; therefore, acute treatment was not required. Separately, no specific restrictions on physical activity or special dietary management were imposed after surgery. Although vigorous exercise is generally discouraged in patients with this condition, the patient was unable to engage in strenuous activity due to limited neck mobility and a restricted field of vision. Furthermore, unlike hepatic-type GSD, there were no issues with blood glucose regulation, and a regular diet was provided.

A previous study^1)^ reported that muscle-type PHK deficiency exhibits 3 clinical phenotypes: (i) juvenile and adult-onset exercise intolerance, (ii) delayed onset of progressive muscle weakness, and (iii) neonatal-onset floppy infant syndrome. The patient had not been diagnosed with GSD type IXd before surgery and had undergone several surgeries. However, upon reviewing his medical records, we found that the preoperative blood tests at the time of his left TEP at the age of 57 years showed a CK level of 608 U/L, suggesting that he had already developed the disease at that time. It is possible that phenotypes (i) and (ii) mentioned above apply to this case, and that the disease was not diagnosed at the time of surgery due to relatively mild symptoms and slow progression.

Excluding our case, only 19 cases of GSD type IXd have been reported over the past 31 years, from 1994 to 2024 (**Table 2**). We reviewed these cases in detail. As this disease is inherited in an X-linked recessive manner, most patients were male; however, females may also develop symptoms to varying degrees depending on the pattern of X-chromosome inactivation.^11)^ We also examined family histories, but most previously reported cases had either no known family history or no records. In some types of GSD, early diagnosis is possible in countries where they are included in newborn mass screening programs. However, as shown in **Table 2**, GSD type IX tends to be diagnosed later due to its rarity, relatively mild symptoms, and generally slow progression.

**Table 2:** Previous reports

Case	Reporting year	Sex	Age (years)	Age at onset (years)	Family history	Chief complaint	CK increase
Wehner et al.	1994	Male	64	46	n.d	Gait disturbance	+2.0-times normal
Bruno et al.	1998	Male	28	15	No	Exercise intolerance Pigmenturia	+3.6-times normal
Burwinkel et al.	2003	Male	36	6	n.d	Exercise intolerance Pigmenturia, cramps	+6.0-times normal
Wuyts et al.	2005	Male	56	43	No	Myalgia	+1458 IU/L
Ørngreen et al.	2008	Male	50	Childhood	n.d	Exercise intolerance	+400 U/L
Echaniz-Laguna et al.	2010	Male	17	17	No	HyperCKemia	+1000 U/L
Preisler et al.	2012	Male	39	32	No	Myalgia	+332 U/L
		Male	69	64	No	HyperCKemia	+1000 U/L
Andersen et al.	2020	Male	33	33	n.d	Myalgia, fatigue	n.d
Li et al.	2020	Male	25	15	Father	Muscle weakness, Myalgia	+464–2842 U/L
Bisciglia et al.	2021	Male	55	40	Sister	Exercise intolerance, Myalgia	+2659 IU/L
		Female	73	72	Brother	Camptocormia	+260 IU/L
Munekane et al.	2022	Male	16	16	No	Exercise intolerance Myalgia, pigmenturia	+213–2027 U/L
Huang et al.	2022	Male	41	40	No	Myalgia, muscle weakness	+17060 U/L
		Male	31	31	No	Muscle weakness	+9200 U/L
		Male	13	13	No	Muscle weakness	+621 U/L
Mori-Yoshimura et al.	2022	Male	78	71	No	Exercise intolerance	+400–700 IU/L
Wang et al.	2022	Male	66	64	No	Muscle weakness	−85 U/L
Picillo et al.	2024	Male	53	16	Mother	Myalgia, muscle weakness	+3.6-times normal
Our case	2025	Male	61	53	No	Exercise intolerance	+598 U/L

+, elevated creatine kinase (CK); −, no CK elevation; n.d, no data

The mean age at onset was 34.95 years, and 12 of the 20 cases (60%) had adult-onset symptoms. The average time from symptom onset to diagnosis was 10.25 years. In all, 8 of the 20 cases (40%) experienced delays of over 10 years, and 12 cases (60%) had a diagnostic delay of more than 5 years. The most common presenting symptoms were myalgia, exercise intolerance, and muscle weakness. Regarding serum CK elevation, 18 cases showed increased CK levels with a mean of 2315 U/L. The highest reported value was 17060 U/L. Approximately half of the patients had levels exceeding 1000 U/L, and around 78% had levels of over 500 U/L. A case has reported characteristic physical findings such as pseudohypertrophy of the quadriceps and gastrocnemius muscles due to glycogen accumulation and fatty replacement.^11)^ In summary, this disease can be suspected in adults—particularly males—presenting with muscle pain, muscle weakness, and elevated CK levels.

Therefore, when performing surgery in patients with pathological muscle weakness or elevated preoperative CK levels, it is crucial to carefully consider the surgical technique in collaboration with specialists from other departments and conduct more detailed postoperative management than usual, because these patients may have undiagnosed GSD.

## CONCLUSIONS

We successfully managed a patient with GSD type IXd in the perioperative setting. Although the postoperative course was favorable, the disease carries the risk of complications such as rhabdomyolysis. Because the symptoms are often mild, the condition may remain undiagnosed. Therefore, careful surgical planning and perioperative monitoring are essential in patients with unexplained muscle weakness or elevated CK levels.

## DECLARATIONS

### Availability of data and materials

Data sharing is not applicable to this article.
